# Advances of hydrogel combined with stem cells in promoting chronic wound healing

**DOI:** 10.3389/fchem.2022.1038839

**Published:** 2022-11-28

**Authors:** Qirong Li, Dongxu Wang, Ziping Jiang, Rong Li, Tianyi Xue, Chao Lin, Yongzhi Deng, Ye Jin, Baozhen Sun

**Affiliations:** ^1^ Department of Hepatobiliary and Pancreas Surgery, China-Japan Union Hospital of Jilin University, Changchun, China; ^2^ Laboratory Animal Center, College of Animal Science, Jilin University, Changchun, China; ^3^ Department of Hand and Foot Surgery, The First Hospital of Jilin University, Changchun, China; ^4^ School of Grain Science and Technology, Jilin Business and Technology College, Changchun, China; ^5^ School of Pharmacy, Changchun University of Chinese Medicine, Changchun, China

**Keywords:** hydrogel, wound healing, stem cell therapy, diabetes, burn

## Abstract

Wounds can be divided into two categories, acute and chronic. Acute wounds heal through the normal wound healing process. However, chronic wounds take longer to heal, leading to inflammation, pain, serious complications, and an economic burden of treatment costs. In addition, diabetes and burns are common causes of chronic wounds that are difficult to treat. The rapid and thorough treatment of chronic wounds, including diabetes wounds and burns, represents a significant unmet medical need. Wound dressings play an essential role in chronic wound treatment. Various biomaterials for wound healing have been developed. Among these, hydrogels are widely used as wound care materials due to their good biocompatibility, moisturizing effect, adhesion, and ductility. Wound healing is a complex process influenced by multiple factors and regulatory mechanisms in which stem cells play an important role. With the deepening of stem cell and regenerative medicine research, chronic wound treatment using stem cells has become an important field in medical research. More importantly, the combination of stem cells and stem cell derivatives with hydrogel is an attractive research topic in hydrogel preparation that offers great potential in chronic wound treatment. This review will illustrate the development and application of advanced stem cell therapy-based hydrogels in chronic wound healing, especially in diabetic wounds and burns.

## 1 Introduction

Wound formation occurs due to physical or chemical factors or skin and soft tissue damage caused by medical and physiological conditions (Eming et al., 2014). Wound healing results from the interaction of a series of biomolecules and stem cells and is considered one of the most complex dynamic physiological processes in the human body (Hadisi et al., 2018). An acute wound is defined as a skin injury due to trauma or surgery that usually heals in an orderly and timely process without complications (Lazarus et al., 1994). Chronic wounds usually do not regain their functional integrity within 3 months or even show a tendency to heal (Malone-Povolny et al., 2019). They are more likely to be caused by continuous stimulation and not only to heal slowly but also relapse. The Wound Healing Society classifies chronic wounds into four categories, depending on their cause: pressure ulcers, venous ulcers, arterial insufficiency ulcers, and diabetic ulcers (Kirsner, 2016). Compared with the normal wound healing process, the causes and symptoms of chronic wound healing are more complex and severe (Martin and Nunan, 2015). Therefore, chronic wound healing is a significant challenge in modern medicine. There is an urgent need to conduct relevant research on promoting chronic wound healing. Stem cells are a special type of cell with extensive self-renewal ability and further differentiation ability, which can respond to injury and repair tissues by proliferating and differentiating (Watt and Driskell, 2010). They can not only differentiate into different functional cells and promote the whole regeneration process but also stimulate and promote tissue regeneration by secreting functional growth factors (Nourian Dehkordi et al., 2019). In addition, they can regulate angiogenesis, remodeling, cell recruitment, and the immune system (Stappenbeck and Miyoshi, 2009). Therefore, different types of stem cells play important roles in wound healing, including epidermal and dermal stem cells, mesenchymal stem cells (MSCs), endothelial progenitor cells (EPCs), and hematopoietic stem cells (HSCs) (Kucharzewski et al., 2019). Importantly, stem cell therapy shows excellent potential for promoting wound repair.

Many chronic wounds require treatment with wound dressings, which prevent pollution and repeated damage, protect against compression hemostasis and swelling, apply drugs to the wound, and remove necrotic tissue (Zeng et al., 2022). Hydrogel is considered an ideal material for chronic wound dressing because of its three-dimensional structure, good permeability and biocompatibility, easy cleaning, and provision of a moist environment conducive to wound repair (Tavakoli and Klar, 2020). Numerous studies have shown that hydrogel is a new biomaterial with broad application prospects in wound healing (Wang et al., 2021). Hydrogels commonly used in chronic wound healing include polypeptide-based gelatin, silk fibroin, fibrin, polysaccharide-based chitosan, hyaluronic acid, and alginate. Hydrogels are formed through the crosslinking of hydrophilic polymer chains in water (Lu et al., 2018). The many methods for preparing hydrogel include electrostatic interaction and covalent chemical bond crosslinking (Su et al., 2021). Traditional hydrogels lack strength and are prone to permanent breakage, and their internal structure is simple and lacks specialized functions (Zhang and Khademhosseini, 2017). Therefore, hydrogels currently in use are typically modified by adding chemical or molecular substances or optimizing mechanical properties. According to the different needs of chronic wounds, hydrogels with anti-inflammatory, antioxidant, pro-angiogenic, antibacterial, hypoglycemic, heat-sensitive, and even multifunctional properties have been produced.

The specialized structure of hydrogels allows for different functional polymers or bioactive substances to be incorporated to promote wound healing (Wang et al., 2021). An area of rapid growth is combining hydrogels with stem cell-based therapies that load stem cells or stem cell derivatives into hydrogels to promote wound healing (Las Heras et al., 2020). This review summarizes the application of different types of hydrogel-based stem cell therapy to treat chronic wounds, including those formed by diabetes and deep burns.

## 2 Chronic wounds

**FIGURE 1:**
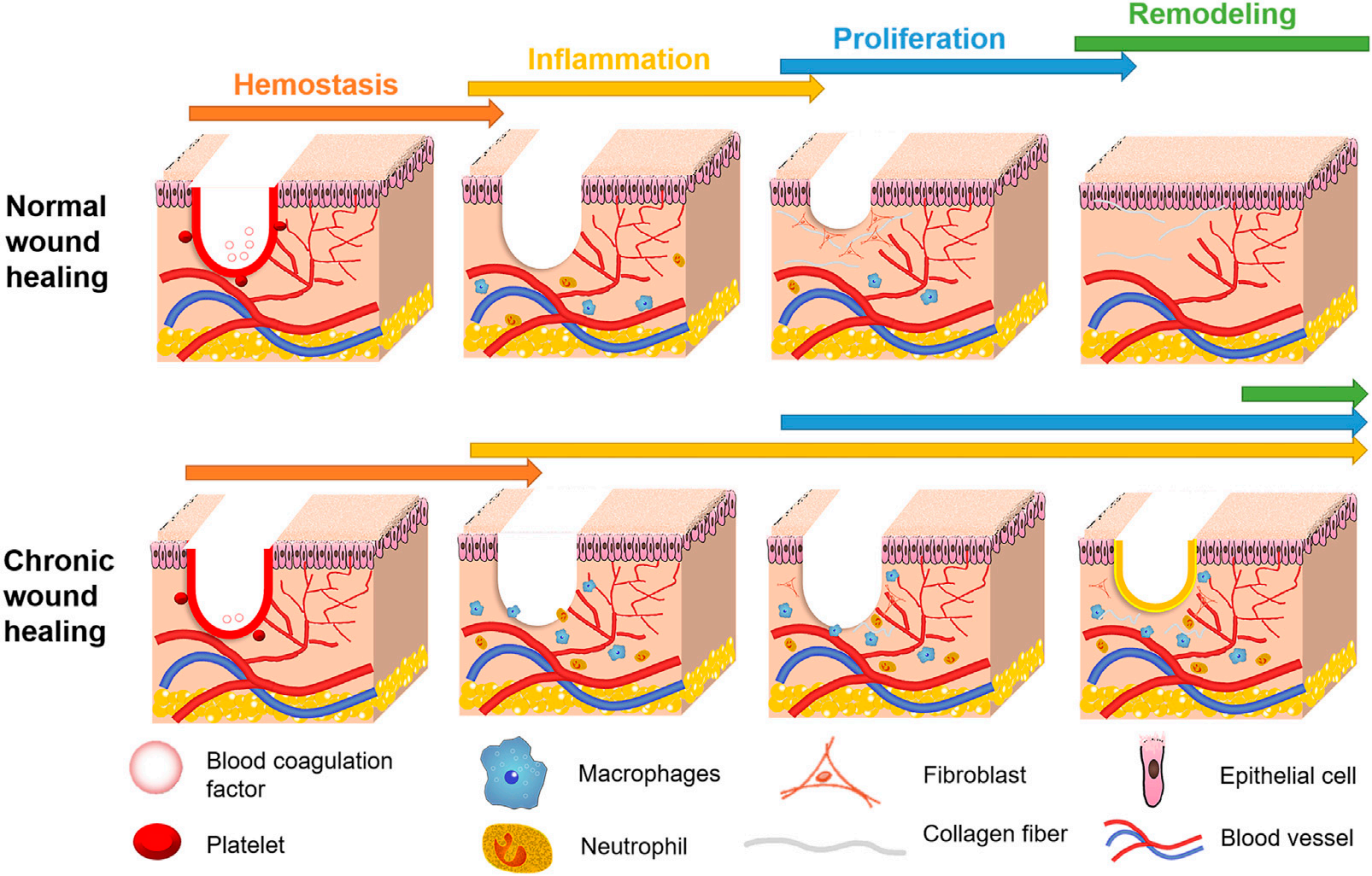
Comparison of healing process between chronic wound and normal wound. The U-shaped red line in wound area indicates that the wound is in a hemostatic stage, while the yellow line indicates that the wound is not healing and is in an inflammatory state.

When the skin or surface soft tissue is damaged by trauma, injury, burn, ulcer, surgery, chronic disease, or inflammation, it usually hinders basic physiological functions in the human body and requires repair through a process called wound healing (Eming et al., 2014). When the wound healing process is disturbed, it can slow healing and cause pathological changes, forming chronic wounds (Richmond and Harris, 2014). Most chronic wounds are serious skin and tissue injuries, which are difficult to heal, and cause the body to receive external stimuli directly (Wilkinson and Hardman, 2020). In addition, slow wound healing can lead to poor patient resistance, malnutrition, septicemia, and other complications (Guo and Dipietro, 2010). Furthermore, chronic wounds that repeatedly trigger inflammatory responses can lead to immune and metabolic disorders and further damage vascular structural integrity and tissue regeneration (Malone-Povolny et al., 2019). At present, hydrogels are recognized as biological materials with properties well suited to wound dressing. Normal wound healing involves the orderly progression of hemostasis, inflammation, proliferation, and remodeling (Guo and Dipietro, 2010). However, chronic wounds do not complete this process effectively, as shown by prolonged hemostasis or inflammation, resulting in a failure of proliferation and remodeling (Figure 1).

### 2.1 Factors affecting chronic wound healing

The main stages of subdivision that occur during wound healing include hemostasis, collagen deposition, stromal remodeling, angiogenesis, re-epithelialization, and scar formation (Laurens et al., 2006). Factors that can cause wound healing to fail include hyperglycemia, repeated tissue damage (Randeria et al., 2015), long-term or excessive inflammation, persistent infection, and attenuation of microbial, dermal, and epidermal responses in repair stimulation (Frykberg and Banks, 2015). Skin structure can be divided into three layers: the *epidermis* (epithelium), dermis, and subcutaneous layer (Vijayavenkataraman et al., 2016). During hemostasis, blood vessels constrict, and platelets accumulate on the subcutaneous surface (Kharaziha et al., 2021). At this stage, fibrin plays a role in wound healing by regulating hemostasis and internal environmental balance (Heher et al., 2018). Fibroblasts in the subcutaneous tissue proliferate during the cell proliferation phase to form contractile granulation tissue and produce collagen deposition (Li and Wang, 2011). Proliferating fibroblasts reshape the extracellular matrix (ECM), while endothelial cells proliferate, migrate, and recombine to form new blood vessels (Tracy et al., 2016). In the re-epithelialization stage, epidermal stem cells proliferate to rebuild the *epidermis* (Ben Amar and Wu, 2014). At the same time, the body will undergo an immune response, where macrophages, neutrophils, and other immune cells participate in the wound healing process. Therefore, the cells recruited to the wound contribute to wound healing through proliferation, differentiation, or secretion of regulatory substances.

In addition, certain cytokines play an important role in the wound healing process. Macrophages and neutrophils secrete inflammatory cytokines to slow wound healing (Krzyszczyk et al., 2018). For example, vascular endothelial (VEGF), fibroblast (FGF), and platelet-derived (PDGF) growth factors affect hemostasis and angiogenesis (Shen et al., 2016). Moreover, hepatocyte growth factor (HGF) contributes to the re epithelialization of injured tissues (Dally et al., 2017), while both FGF and epidermal growth factor (EGF) regulate the re-epithelialization process (Seeger and Paller, 2015). HGF regulates re-epithelialization by binding to and activating the MET receptor tyrosine kinase (Chmielowiec et al., 2007). The re-epithelialization regulators HGF, FGF, and EGF are also ligands of receptor tyrosine kinases. Receptor tyrosine kinase activation usually stimulates the migration, proliferation, and survival of keratinocytes (Schafer and Werner, 2007). Moreover, the proliferation-promoting transforming growth factor-β (TGF-β) regulates the transcriptional regulator SMAD3 and influences the epithelial-mesenchymal interaction (Werner and Alzheimer, 2006).

MSCs can promote wound healing, especially in the treatment of chronic wounds (Rodgers and Jadhav, 2018). MSCs are thought to migrate to damaged tissue and release growth factors such as EGF, FGF, PDGF, and TGF -β, VEGF, insulin-like growth factor-1, angiopoietin-1 (ANGPT1), and stromal cell-derived factor-1 all influence fibroblast and endothelial cell development (da Silva Meirelles et al., 2009). Moreover, dendritic (DC) and natural killer (NK) cells mediate the tissue injury immune process in addition to inhibiting lymphocytes, and MSCs interact with macrophages and neutrophils to produce important regulatory effects (Seno et al., 2009). For example, MSCs participate in immune regulation through the PD-1:PD-L1/2 pathways (Corcione et al., 2006). In addition, epidermal stem cells, embryonic stem cells (ESCs), and induced pluripotent stem cells (IPSs) may also promote the chronic wound healing process.

### 2.2 Diabetes and burn wounds

Some conditions and diseases, such as diabetes and deep burns, can significantly affect the rate of wound healing and even cause the formation of chronic wounds. Diabetes is a metabolic disease characterized by hyperglycemia, which can cause chronic damage and dysfunction of various tissues, especially the eyes, kidneys, heart, blood vessels, and nerves (American Diabetes, 2014). Diabetic patients often have a weakened immune system and suffer long-term complications, including chronic wounds. The main features of diabetic wounds are imbalanced inflammatory reaction, increased risk of infection, and insufficient angiogenesis, which are usually affected by complex factors such as immune deficiency, dysfunction, and local infection (Wang et al., 2021). The average wound healing process lasts 12–13 months, and the possibility of recurrence is very high, which seriously affects the comfort and health of patients and increases the cost and difficulty of treatment (Richmond et al., 2013). The wound healing process in people with diabetes is stagnant at the inflammatory stage, characterized by elevated levels of pro-inflammatory cytokines, protease and reactive oxygen species (ROS), and cell dysfunction (Frykberg and Banks, 2015). ROS induces the expression of serine proteases and matrix metalloproteinases (MMPs), leading to ECM and growth factor degradation, further inhibiting wound healing and promoting an inflammatory response (Demidova-Rice et al., 2012). In addition, their accumulation of advanced glycation end products (AGEs) can induce ROS production and induce nuclear factor kappa B (NF-κB) activation through binding to the receptor for advanced glycation endproducts (RAGE) expressed in various skin cells, including keratinocyte, fibroblast, dendritic, endothelial, and mononuclear cells, leading to pathological gene expression (Lohwasser et al., 2006).

The severity of a burn victim’s injury depends on the wound’s depth and the body surface area affected. From minor to the most severe burns requiring the highest level of intensive care and surgery, chronic wounds are considered deep burns (Wasiak et al., 2013). Deep burns involve deeper skin structures, such as blood vessels, nerves, and hair follicles, causing considerable pain and taking a long time to heal (Zhang et al., 2015). How the burns are treated also influences their wound healing time and affects their risk of infection. Superficial burns may develop into deeper wounds if they are dry or infected. The malignant transformation of burns can lead to tissue ischemia, cytokine environment disturbance, and free radical damage, causing further protein degeneration and necrosis (Singh et al., 2007). Common complications from burns caused by abnormal wound healing include a hypertrophic scar, post-burn contracture, and non-healing, where increased smooth muscle alpha-actin (α-SMA) and collagen levels due to myofibroblast activation are markers of hypertrophic scar formation (Hall et al., 2017). Therefore, given the serious impact of chronic wounds caused by diabetes and burn on patient recovery and health, the development of new diabetic wounds and burn dressings are of particular importance.

## 3 The application of hydrogel combined with stem cells in the treatment of chronic wounds

Numerous methods for treating chronic wounds have been proposed that each target different stages in the wound healing process. These methods include different types of dressing, delivery of cytokines and growth factors, cell therapy, and application of electrical or mechanical stimulation (Frykberg and Banks, 2015). Among them, using artificial construction biomaterials as dressings that promote the wound healing process is considered one of the most effective strategies. Given this, many different types of biomaterial-based wound dressings have recently been developed to simulate the skin’s microenvironment (Bhardwaj et al., 2017). Among them, hydrogels with bionic structure and physical properties have been widely used in wound healing (Xie et al., 2015). Hydrogel has high biocompatibility, encapsulating all types of cells and biological macromolecules that are then released under various external conditions (Naahidi et al., 2017). Hydrogel is an insoluble polyurethane polymer with high hydrophilic content. Its hydrophilicity is determined by the cross-linking degree of polar functional groups. The high porosity and large specific surface area of hydrogels also enable them to retain water and transport materials due to their 3D porous network structure (Hao et al., 2021). Based on their rheological properties, hydrogels are viscoelastic in nature and the hydrogel will not stick to the wound, which can be conveniently smeared and removed without causing secondary damage (Wang et al., 2021). This versatility makes hydrogel a promising biomaterial for delivering therapeutic molecules and indicates that hydrogel dressings incorporating stem cell therapy have good prospects in chronic wound healing. Moreover, different hydrogels have different characteristics that can improve stem cell delivery to the wound depending on their properties (Table 1).

**TABLE 1:** The main raw materials and characteristics of hydrogel wound dressing.

	Type	Material	Characteristic	Origin	Reference
Natural substanc-es	Polysacc-haride	Alginates (alginic acid)	High biocompatibility; low cost; can undergo gelation with divalent cations under mild conditions suitable for encapsulation of bioactive molecules and living cells	Algae cell walls	Sun et al. (2016)
		Chitosan	Antibacterial activity; the positive charge of protonated amino group of chitosan can interacts with the negative charge molecules on the surface of bacterial cells; good biocompatibility; biodegradability and are good cell and drug carriers; poor mechanical properties	Deacetylated chitin from crustaceans	Tian et al. (2021)
		Cellulose	3D fibrous; porous microstructure; high water content (98–99%); water uptake and water retention capacity; high mechanical strength and flexibility; good permeability; biodegradability; biocompatibility	Algae/plant cell walls or secreted by bacteria	Alven and Aderibigbe, (2020)
		Hyaluronic acid	High water retention capacity; elasticity; vital element of viscoelastic tissues; not adhesive for cells	ECM component	da Silva et al. (2017)
		Sodium alginate	Excellent biocompatibility; potential hemostatic biomaterials; ease of gelation; high hydrophilicity; biodegradability	Kelp, seaweed	Zhenkun Zhang et al. (2021)
		Gum arabic	Antibacterial and antioxidant activities	Arabic	Ahmad et al. (2019)
	protein	Collagen	Inhibit bacterial growth; prolonged inflammatory response; low immunogenicity; biocompatibility; similarity to the natural ECM	ECM component	Garg et al. (2014)
		Elastin	Inherent biocompatibility; biodegradability; weak mechanical; adhesive properties	ECM component	Rodriguez-Cabello et al. (2018)
		Fibrin and Fibrinogen	The similarity properties of physiological fibrin; can activate the coagulation cascade; extensibility	Blood clotting protein	Murphy et al. (2017)
		Gelatin	Derivative of collagen; excellent gelling properties; biocompatible; biodegradable; poor mechanical properties	Hydrolyzed collagen	Dong et al. (2018)
		Silk fibroin	Biocompatibility; tunable mechanical properties and degradation rate; limited inflammation-inducing properties	Silk	Han et al. (2021)
Artificial synthetic materials	PAA	Poly (acrylic acid)	Biocompatible; biodegradable; enhance cell adhesion and proliferation	Synthesized from acrylic acid	Rasool et al. (2019)
	PEG	Poly (ethylene glycol)	Can leads to cytoplasmic spreading and the formation of cellular networks that improves cellular delivery and extends survival time of cells; can enhance the mechanical strength, degradation rate and stability of hydrogel	Synthesized From ethylene oxide	Guerra et al. (2017)
	PLGA	Poly (lactic‐co‐glycolic acid)	Biocompatible; biodegradable; its hydrolysis products can be uptaken in the cellular metabolic pathway	Synthesized from glycolic acid and lactic acid	Lee et al. (2007)
	Polypepti-des	Various amino acid sequences	Self-assembled supramolecular physical gel; biodegradability; target specificity; less side effects; injectable; amphiphilic; safety	Amino acid chains bound covalently (by peptide bonds)	Cai et al. (2020)
	Pluronic F-127	Polyethylene-polypropylene glycol	Unique heat-sensitive properties; injectable; biodegradable; porous structure; mild inflammatory property; the ability to absorb the secretions from the wound surface	Synthesized from ethylene and propylene glycol	Jiao et al. (2021)

MSCs are a type of pluripotent stem cell with all the attributes commonly associated with stem cells, including the ability to self-renew and multidirectional differentiation (Chen et al., 2022), found in the bone marrow, adipose tissue, umbilical cord, and placenta (Lin et al., 2019). MSCs offer great potential in treating various diseases, especially those related to tissue injury (Bhardwaj et al., 2017), as shown by numerous studies that have used them to promote chronic wound healing (Varderidou-Minasian and Lorenowicz, 2020). MSCs and MSC-like cells exist in almost all tissues, including bone marrow, muscle, fat, hair follicle, and dermis (Bianco et al., 2008). Moreover, MSCs have the advantages of wide distribution and easy separation and culturing *in vitro* (Shi et al., 2010). However, the realization of clinical treatment requires the delivery of large numbers of stem cells to specific sites in the body with high precision. Due to their low transplantation efficiency, the clinical transformation of MSCs is hard to achieve. Therefore, combining them with biomaterials such as hydrogel has the potential to improve the efficiency and stability of MSCs delivery, enhancing traditional MSC-mediated repair and related cellular functions (Meier et al., 2015). The combination of MSCs and hydrogel has become the leading direction in wound healing research.

### 3.1 Hydrogels based on bone marrow MSCs promote chronic wound healing

**FIGURE 2:**
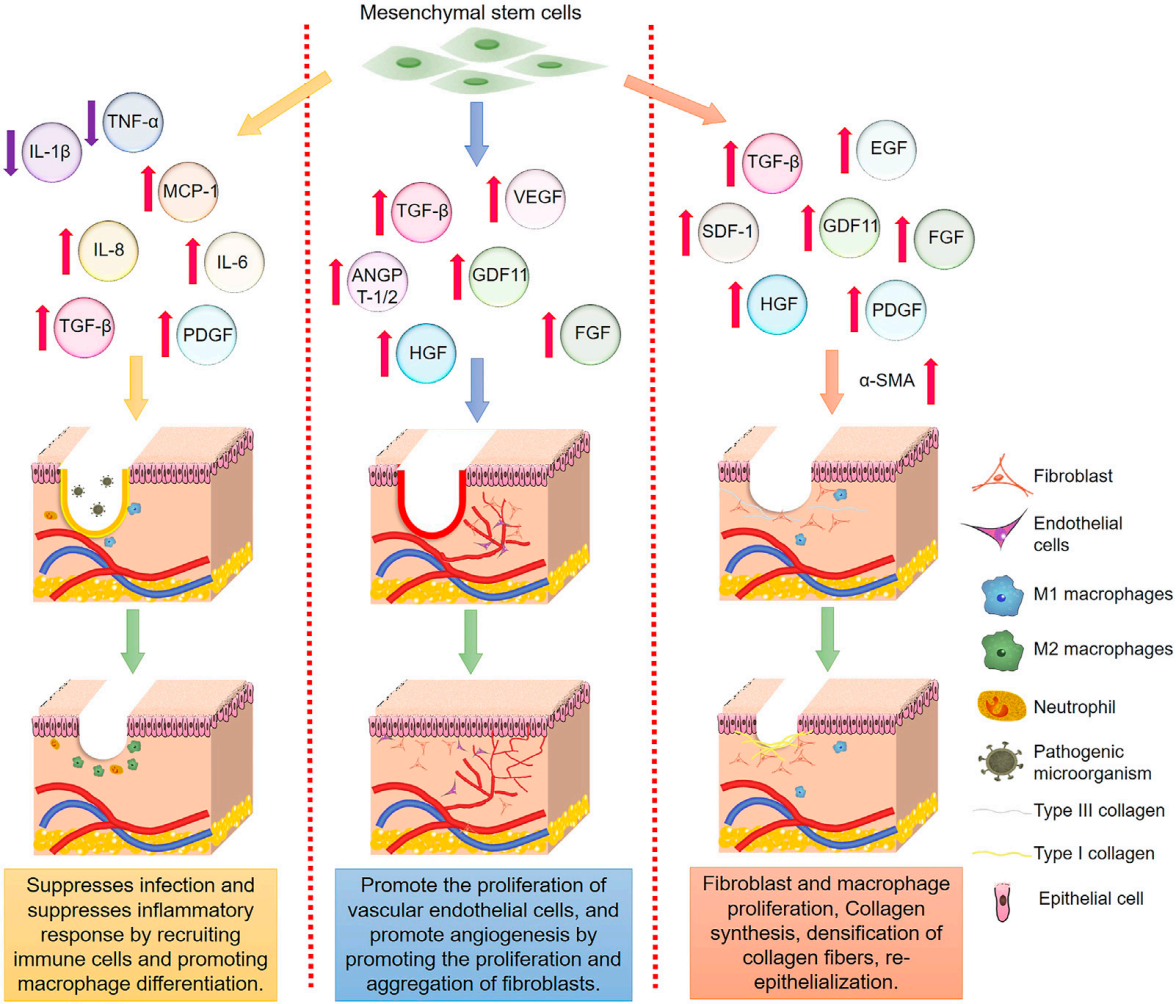
Important mechanism of stem cells regulating wound healing.

Bone marrow mesenchymal stem cells (BMSCs) are a type of adult stem cell originating from the mesoderm, which can differentiate into various mesenchymal tissues, such as bone, cartilage, adipose, bone marrow, and hematopoietic (Wu et al., 2018). MSCs regulate the wound repair process by differentiating into several stromal or damaged cell types and interacting with many types of tissue and immune cells through bioactive factors, including fibroblasts, endothelial and epithelial cells, macrophages, neutrophils, and lymphocytes (Shi et al., 2010) (Figure 2).

The Detachable hybrid microneedle depot (d-HMND) made of gelatin methacryloyl (GelMA) hydrogel is an efficient drug delivery vehicle that can minimize drug dose and can degrade stably for long-term release delivery cells. The d-HMND has been proposed for BMSCs delivery and demonstrated *in vitro* and *in vivo* that MSCs delivered by hydrogel can promote mouse full skin thickness excisional wound regeneration by secreting VEGF (Lee et al., 2020). Besides, *in vitro* experiments showed that BMSCs encapsulated in a polyethylene glycol diacrylate (PEGdA) and thiogelatin polyethylene glycol (Gel-PEG-Cys) crosslinked hydrogel were shown to have a beneficial effect on wound healing and the antibacterial loaded hydrogel enhanced the proliferation, chemotaxis and haptotaxis of stem cells (Guerra et al., 2017). A fibrin hydrogel carrier was designed to enhance the angiogenesis and anti-inflammatory ability of the encapsulated BMSC globules and promote the secretion of VEGF and prostaglandin E2 (PGE2) and wound healing in three-dimensional endothelial human skin equivalent model (Murphy et al., 2017). In addition, thermosensitive chitosan (HBC) modification to create an eLHBC hydrogel coated in BMSCs was found to have adhesiveness and antibacterial activities and facilitate wound healing by promoting fibroblast migration and secretion of VEGF and FGF in the incisions of rats skin (Tian et al., 2021). Furthermore, BMSCs combined with a novel biocompatible heat-sensitive hydrogel have been shown to enhance α-SMA expression in full-thickness wound of mouse model, significantly promoting wound healing, epithelial cell proliferation, re-epithelialization, and collagen deposition (Lei et al., 2018). In addition, a fibrin hydrogel containing 0.7 × 10^6^ BMSCs was implanted subcutaneously in rats and found to promote wound healing and repair and interact with VEGF to promote endothelial cell proliferation and differentiation, potentially promoting angiogenesis in the injured areas (Tan et al., 2021). A thinned silk nanofiber hydrogel encapsulating BMSCs and injected into the SD rats wound site has been shown to accelerate wound healing by promoting the up-regulation and secretion of ANGPT1 and HGF. And shear thinning hydrogels can protect BMSCs and optimize their behavior during injection. (Li J. et al., 2021). Furthermore, delivery of BMSCs extracellular vesicles (EVs) loaded with miR-29b-3p in a bilayered thiolated alginate/PEG diacrylate (BSSPD) hydrogel showed that hydrogels can sequentially release EVs and miR-29b-3p can rapidly heal wounds and reduce scar formation by inhibiting collagen type I alpha 1 chain (Col1A1) expression in the full-thickness skin defect model of rats and rabbit ears (Shen et al., 2021). Therefore, BMSCs are the most common type of stem cell used to promote wound healing, as they can promote cell proliferation and angiogenesis and inhibit inflammation.

### 3.2 Hydrogels based on adipose MSCs promote chronic wound healing

Adipose mesenchymal stem cells (ADSCs) are pluripotent stem cells derived from adipose tissue that can be induced to differentiate into various cell types under specific conditions, including adipose, bone, cartilage, islet β, and cardiac muscle (Miana and Gonzalez, 2018). Recently, numerous studies have applied ADSCs to wound healing, where they act on fibroblasts, macrophages, and skin cells by secreting growth factors, including growth differentiation factor 11 (GDF11) and TGF-β, and regulating the immune response, cell proliferation, and angiogenesis during wound healing (Mazini et al., 2020).

A short peptide (GV8) hydrogel can self-assemble and maintain the activity of delivery cells under mild physiological conditions. GV8 peptide hydrogel containing ADSCs secretome has been shown to have good potential in full-thickness excisional wound healing C57/BL6 mice model (Hiew et al., 2021). Here, ADSCs secrete factors that regulate angiogenesis and endothelial cell migration (thymosin beta 4 X-linked [TMSB4X], gremlin 1 [GREM1], and extracellular matrix protein 1 [ECM1]), metalloproteinase inhibition (tissue inhibitors of metalloproteinases 1 [TIMP1]), cell proliferation (EGF containing fibulin extracellular matrix protein 1 [EFEMP1]), immune cell proliferation and morphogenesis (colony-stimulating factor 1 [CSF1]), and growth factor interaction (latent transforming growth factor-beta binding protein 1 [LTBP1]) (Hiew et al., 2021). In addition, platelet-rich plasma hydrogel can provide a stable physical framework and promote the potential of ADSCs *in vitro* and *in vivo*, increasing their expression of ANGPT1 and angiopoietin 2 (ANGPT2) and promoting male athymic rats wound healing (Samberg et al., 2019). Similarly, a biomimetic pullulan collagen hydrogel scaffold can deliver ADSCs into the splinted murine wounds environment and enhance their stem cell properties by increasing the expression of octamer-binding transcription factor 4 (Oct4). Moreover, ADSCs accelerate wound healing by significantly enhancing the expression of multiple factors, including stromal cell-derived factor-1 (SDF-1), monocyte chemoattractant protein-1 (MCP-1), fibroblast growth factor 2 (FGF-2), insulin-like growth factor 1 (IGF-1), VEGF-a, endoglin (ENG), HGF, and ANGPT1 (Garg et al., 2014). In addition, UV crosslinked biodegradable hydrogel containing ADSCs can be used as a the dermal layer of a bilayer skin substitute and promote the formation of blood vessels and increase the number of endothelial cells (Eke et al., 2017). Moreover, an adhesive hydrogel consisting of alginate, Arabia gum, and calcium ions has been synthesized and shown excellent mouse wound healing abilities after combined use with 1 × 10^6^ mouse ADSCs (Sun et al., 2016). Furthermore, a hydrogel loaded with the secretory body from the human ADSC line HATMSC2 was found to contain high levels of interleukin-8 (IL-8) and MCP-1, and relatively high expression of the pro-angiogenic microRNAs miR210, miR126, and miR296, indicating that it has good potential in chronic wound treatment (Kraskiewicz et al., 2021). Moreover, the ADSC-derived exosomes applied within an alginate-coated hydrogel significantly promoted wound closure, collagen synthesis, and wound angiogenesis in the full-thickness excisional wound of rat model (Shafei et al., 2020). At present, stem cell therapies based on ADSCs are gradually emerging, and the combination of ADSCs and hydrogel has gradually become mainstream in wound healing research.

### 3.3 Hydrogels based on other types of stem cells promote wound healing

Umbilical cord mesenchymal stem cells (UCMSCs) are a type of multifunctional stem cell that exists in the neonatal umbilical cord tissue and can differentiate into many types of tissue cells. Studies have shown that functional injectable thermosensitive chitosan hydrogel-coated 5 × 10^6^ human UCMSCs can promote SD rats skin wound healing and inhibit wound inflammation by reducing tumor necrosis factor-α (TNF-α) and interleukin 1 beta (IL-1β) protein levels (Xu et al., 2019). Another study constructed a biodegradable, dual-sensitive hydrogel-coated human UCMSC-derived exosomes that inhibit inflammatory responses and promote female SD rats wound healing and skin regeneration by promoting collagen deposition and regulating TNF-α and IL-1β expression (Li Q. et al., 2021). In addition, human UCMSC-derived exosomes in a silk fibroin and sericin composite hydrogel can promote vascular growth and inhibit the inflammatory response, promoting C57BL/6J mice wound healing (Han et al., 2021). Furthermore, sodium alginate/collagen (SA/Col) hydrogel is injectable, biodegradable, and has low immunogenicity, which can promote the retention and survival of UCMSCs *in vivo*. Studies have shown that a SA/Col hydrogel containing human UCMSCs significantly upregulates TGF-β1 expression, accelerates keratinocyte maturation, inhibits inflammatory responses by inhibiting NLRP3 signaling, and promotes wound healing and skin regeneration (Zhang Z. et al., 2021).

Human amniotic mesenchymal stem cells (hAMSCs) derived from placental amniotic tissue are adult stem cells with apparent plasticity and multidirectional differentiation potential. Studies have shown that hAMSCs are suitable for skin tissue engineering and regenerative medicine (Farhadihosseinabadi et al., 2018). Moreover, study have shown that an acellular dermal matrix hydrogel decorated with carbon nanodots can enhance chronic wound healing by transporting hAMSCs to clear ROS and upregulate collagen I antibody expression in adult Wistar male rats (Bankoti et al., 2020).

Compared with IPSs or ESCs, tissue-specific mature stem cells such as MSCs are considered more acceptable and safer and have more potential for medical applications (Sharma et al., 2021). Therefore, most studies combining stem cell therapy with hydrogel materials to promote wound healing have used MSCs or their derivatives. However, some studies have shown that functional tissue regeneration can be achieved using mesenchymal cells derived from human ESCs in a hydrogel matrix (Hwang et al., 2008), and ESCs may offer another promising direction in wound healing research. While UCMSCs and hAMSCs are rarely used at present, they also have beneficial effects on promoting wound healing, and like other unstudied stem cell types, would benefit from additional research and development.

## 4 The application of hydrogel combined with stem cells in the treatment of diabetes and burn chronic wounds

**FIGURE 3:**
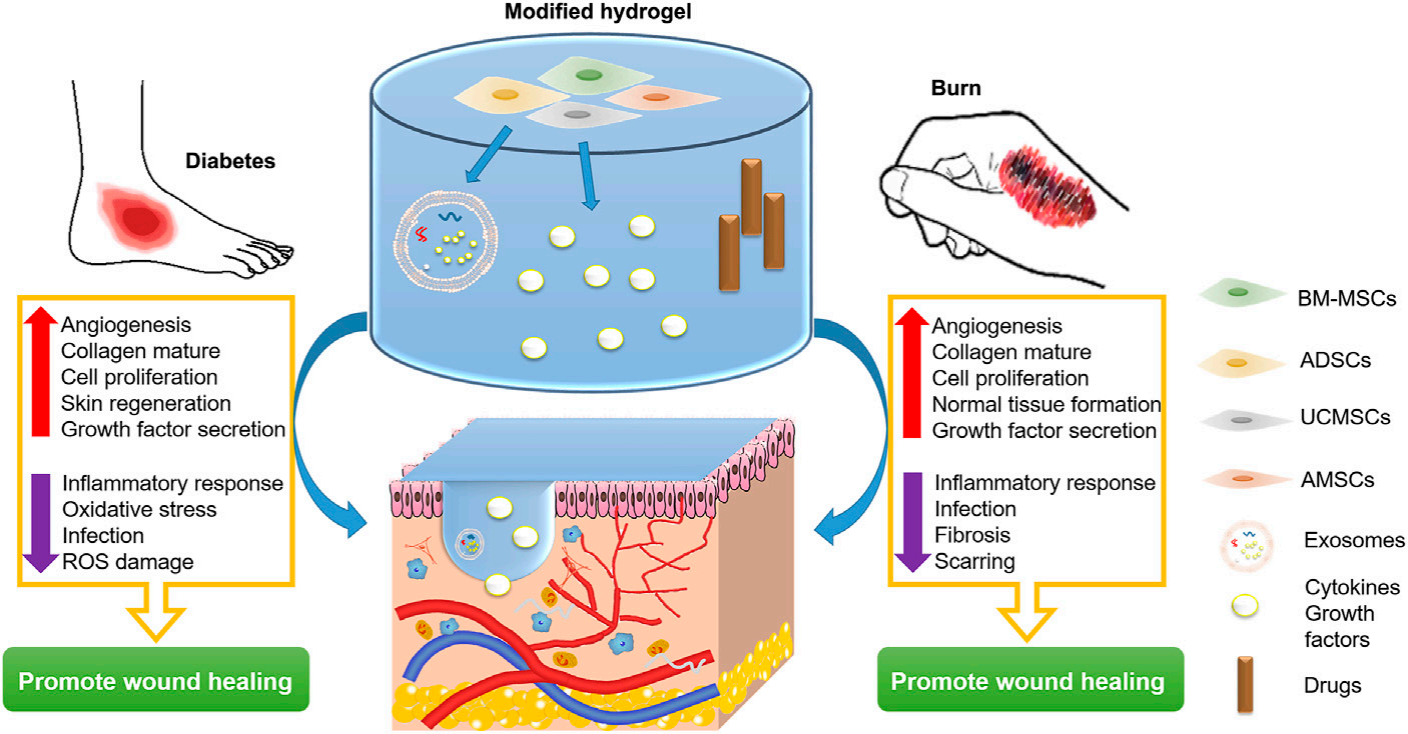
Strategy of hydrogel stem cells for diabetes wounds and burn treatment.

Diabetic wounds no longer have the normal skin regeneration function and are difficult to heal through normal physiological processes (Greenhagen et al., 2019). They usually occur on the limbs, especially the feet, and are prone to infection and recurrence (Krishnan et al., 2007). Severe full-thickness burns involve all skin structures, including blood vessels, nerves, and hair follicles, and may also involve underlying structures such as muscle and bone. Burns are the main cause of extensive body surface hypertrophic scarring (Markeson et al., 2015). Wound dressing with hydrogel stem cells can promote wound healing by secreting cell growth factors and exosomes and cooperating with drugs (Figure 3).

### 4.1 Hydrogels based on stem cells promote diabetic wound healing

Diabetic chronic wounds are characterized by unbalanced inflammatory responses, oxidative stress effects, hyperglycemia, absence of angiogenesis, and a high risk of bacterial infection (Wang et al., 2021). Therefore, hydrogel-based stem cell therapy as a new biomaterial has attracted much attention in promoting diabetic wound healing because of its water absorption, permeability, biocompatibility, and drug loading ability, which can control drug release and provide a stable and suitable growth environment for chronic wounds (Cascone and Lamberti, 2020). Introducing stem cells or bioactive substances derived from stem cells into hydrogels can regulate the chronic wound microenvironment and accelerate wound healing in diabetic patients.

A new type of chitosan polyurethane hydrogel membrane (HPUC) with excellent antibacterial and hemostatic activity and bioabsorbability has been developed, which promotes bone healing and reduces inflammation by transplantation of BMSCs in rats with diabetes mellitus (DM) (Viezzer et al., 2020). Another study used a self-healing hydrogel containing 2×10^5^ BMSCs to release TGF-β1, VEGF, and basic FGF, to inhibit the activation of M1 macrophages and promote the activation of M2 macrophages, aiding late wound healing of diabetic foot ulcers in SD rats (Bai H. et al., 2020). It has been reported that a hydroxyapatite/chitosan composite (HAP-Cs) hydrogel coated in miR-126–3p overexpressed MSC-derived exosomes that can activate angiogenesis by targeting the mitogen-activated protein kinase (MAPK)/extracellular signal-regulated kinase (ERK) and phosphoinositide 3-kinase (PI3K)/protein kinase B (AKT) signaling pathways and promote diabetic SD rats wound healing (Li et al., 2016). Other studies have shown that MSCs EVs bind to porcine submucosal hydrogel materials through peptides and promote the proliferation and migration of cells in diabetic rats full-thickness skin wounds by activating the wingless/integrated (Wnt)/β-catenin pathway and promoting angiogenesis by activating the hypoxia-induction-factor-1 α (HIF-1α)/VEGF pathway (Ma et al., 2022). Another study showed that loading ADSCs on the gelatin sericin (GS) hydrogel scaffold inhibits ROS damage and promotes wound angiogenesis in Wistar rat diabetic ulcers. GS hydrogels contain laminin, an endothelial basal protein that can improves angiogenesis (Tyeb et al., 2020). An injectable Gel-PEG hydrogel system was used to transport 3 × 10^5^ ADSCs to diabetic wounds, significantly accelerating wound healing by reducing inflammatory cell infiltration and enhancing neovascularization in a full-thickness excisional wound model in db/db diabetic mice (Dong et al., 2018). Furthermore, ADSCs precultured in hyaluronidase-based spongy hydrogel increased numbers of intraepidermal nerve fibers (IENF), promoting the growth of new nerves, reducing the number of macrophages on the wound surface, and promoting the transition from the inflammatory to proliferative stage of the wound surface, promoting the healing of diabetic mice full-thickness wounds (da Silva et al., 2017). In addition, ADSC exocrine loaded on an antibacterial hydrogel wound dressing promoted wound healing in DM rats by preventing infection, fibroblast proliferation, and granulation tissue formation (Shiekh et al., 2020). Another study developed an injectable, self-healing, and antibacterial polypeptide-based FHE hydrogel that stimulates the release of exosomes from reactive ADSCs and promotes human umbilical vein endothelial cell proliferation, migration, and angiogenesis, co-promoting chronic wound healing and complete skin regeneration (Wang et al., 2019). Besides, a clinical study exploring the potential of hydrogel-based allogeneic ADSCs sheets for the treatment of foot ulcers in diabetic patients found that 82% of the patients in the treatment group had complete wound closure without adverse effects within 12 weeks (Moon et al., 2019). In addition, studies have shown that the combined use of human UCMSC-derived exosomes and F-127 hydrogel enhances VEGF and TGFβ-1 expression, promoting wound healing in DM rats (Yang et al., 2020). Moreover, an injectable hydrogel composed of collagen and polyethylene glycol with controlled drug release and adhesion, loaded with umbilical cord stem cell factor (SCF), has also been developed to treat diabetic rat wounds, decreasing TNF-α expression and increasing VEGF expression in the SCF treatment group (Zhang L. et al., 2021). In addition, the combined use of Wharton jelly mesenchymal stem cells (WJMSCs) with PF-127 hydrogel and sodium ascorbate phosphate (SAP) promoted skin wound healing in diabetic rats, and the addition of SAP reduced the apoptotic rate of WJMSCs in hydrogel by reducing oxidative stress and mitochondrial damage (Jiao et al., 2021). Furthermore, using thermosensitive hydrogel as a scaffold to increase the implantation of muscle-derived mesenchymal stem cells (MDSCs) and improved db/db genetically diabetic mouse model wound healing (Lee et al., 2007). These findings indicate that using hydrogels loaded with stem cells and their derivatives as dressings is a promising method for treating chronic diabetic wounds (Table 2). Furthermore, future research should focus on treating diabetes using modified hydrogels loaded with stem cell exosomes with different characteristics.

**TABLE 2:** Application of hydrogel combined with stem cells in diabetic wound healing.

Stem cell	Hydrogel	Model	Function	Stage of research	Reference
BMSCs	Chitosan polyurethane hydrogel	Rats	Promote bone healing and reduce inflammation	Preclinical	Viezzer et al. (2020)
BMSCs	N-chitosan and Hyaluronic acid hydrogel	SD rats	Promote secretion of factors to inhibit chronic inflammation	Preclinical	Haotian Bai et al. (2020)
BMSCs	Hydroxyapatite/chitosan composite hydrogel	SD rats	Activate angiogenesis and promote wound healing	Preclinical	Li et al. (2016)
BMSCs	Porcine submucosal hydrogel	Rats	Promote the proliferation and migration of cells and promote angiogenesis	Preclinical	Ma et al. (2022)
ADSCs	Gelatin sericin hydrogel	Wistar rat	Inhibit ROS damage and promote wound angiogenesis	Preclinical	Tyeb et al. (2020)
ADSCs	Gel-PEG hydrogel	Db/db diabetic mice	Reduce inflammatory cell infiltration and enhance neovascularization	Preclinical	Dong et al. (2018)
ADSCs	Hyaluronidase-based spongy hydrogel	Mice	Promote the growth of new nerves, reduce the number of macrophages	Preclinical	da Silva et al. (2017)
ADSCs	Elastomeric antioxidant polyurethane hydrogel	Rats	Prevent infection, fibroblast proliferation, and granulation tissue formation	Preclinical	Shiekh et al. (2020)
ADSCs	Polypeptide-based FHE hydrogel	HUVEC	Promote cell proliferation, migration, and angiogenesis	Preclinical	Tyeb et al. (2020)
ADSCs	Hydrogel sheet	Diabetic patients	Treatment group showed significantly faster complete wound closure	Clinical Trial	Moon et al. (2019)
UCMSCs	F-127 hydrogel	Rats	Enhance VEGF and TGFβ-1 expression	Preclinical	Yang et al. (2020)
UCMSCs	Collagen and polyethylene glycol hydrogel	Rats	Decrease TNF-α expression and increase VEGF expression	Preclinical	Li Zhang et al. (2021)
WJMSCs	PF-127 hydrogel	Rats	Improve dermis regeneration and collagen deposition	Preclinical	Jiao et al. (2021)
MDSCs	Thermosensitiv-e hydrogel PEG-PLGA-PEG	Db/db diabetic mice	improve wound healing and promote the development of the essential cells for wound repair	Preclinical	Lee et al. (2007)

### 4.2 Hydrogels based on stem cells promote burn wound healing

Slow wound healing, infection, pain, and hypertrophic scarring remain significant challenges in burn treatment and research (Wang et al., 2018). Severe burns have limited vascular perfusion, which may lead to excessive scarring. Progressive microvascular damage, edema, arteriolar thrombosis, and necrosis due to impaired tissue perfusion can deepen and enlarge the burn wound (Xue et al., 2018). Hydrogel delivery systems not only control the release of bio-therapeutic products such as stem cells in space and time but also mimic the natural ECM microenvironment (Xue et al., 2018). Considering their ease of application and disassembly, dressing change requirements, cost, and patient comfort (Wasiak et al., 2013), the design and modification of hydrogel dressings make them valuable and beneficial for various applications. Stem cells can secrete and chemotaxis growth factors sustainably, respond to local stimuli, influence the wound microenvironment to promote healing, reduce scar formation, improve skin regeneration, regulate inflammatory responses, and reduce the risk of fibrosis and infection (Abdul Kareem et al., 2021). The combined use of stem cells with hydrogels as a wound dressing or skin substitute—another commonly used treatment for burns—protects large wounds and increases the dermal component of the wound to promote healing (Halim et al., 2010).

Studies have shown that a hyaluronic acid hydrogel system can load ADSCs, increase wound angiogenesis and normal tissue remodeling, and significantly increase the expression of growth factors and cytokines that promote female FVB/NJ mice with deep second-degree burns healing, such as PDGF, SDF-1, haemodiafiltration (HDF), metalloproteinase-2 (MMP2), metalloproteinase-9 (MMP9), ANGPT1, ANGPT2, C-C Motif Chemokine Ligand 2 (CCL2), MCP-1, and VEGF-a (Dong et al., 2020). In addition, delivering ADSCs using polyethylene glycol fibrin hydrogels promoted deep partial thickness burn healing and reduced scar production in porcine models by releasing VEGF and increasing collagen fiber densification (Burmeister et al., 2018). Another study used 3D bio-printing to construct an integral 3D-ADscs/NO hydrogel scaffold to enhance the migration and angiogenesis of human umbilical vein endothelial cells (hUVECs) through VEGF expression and promote severe full-thickness burn wound healing in Balb/c mice (Wu et al., 2021). Moreover, autologous stem cells have been isolated from the adipose layer of surgical debridement burn skin (dsADSCs) that can differentiate into the upper cortex, vascularized dermis, and subcutaneous layer in collagenous PEG fibrin-based bilayer hydrogels, which can be used for deep burn skin allografts (Chan et al., 2012). In other studies, 1 × 10^6^ ADSCs loaded into aloe vera hydrogel promoted angiogenesis and granulation tissue formation in rat burn models by increasing the expression of FGF and TGF-β1, promoting debrided full thickness wound healing (Oryan et al., 2019). Furthermore, enzyme crosslinked gelatin hydrogels have been used to encapsulate hADSCs spheroid, which significantly reduced scar formation and promoted neovascularization by releasing PDGF, VEGF, and FGF in male Wistar rats. Besides, it was found that hADSCs spheroid with hydrogel scaffolds could release more growth factors because they had more cell-cell/cell-ECM interactions (Lu et al., 2020).

Current stem cell therapies specifically applied for burn treatment are still based primarily on ADSCs, with the direct encapsulation of stem cells used. Applying other types of stem cells or stem cell-derived bioactive substances, such as exosomes, to deep burns is an important future research direction.

## 5 Discussion

Chronic wound healing is an important healthcare issue, and research on promoting chronic wound healing is an important area. The treatment of chronic wounds in diabetes and severe burns is also an urgent research topic. Wound dressings used to accelerate wound healing have attracted much attention, and many studies believe that hydrogels are one of the most valuable biomaterials for promoting chronic wound healing due to their unique properties, including biocompatibility, moisture retention, ductility, permeability, and controlled delivery of therapeutic drugs (Zhang et al., 2020). Currently, hydrogels used in most studies are loaded with functional bioactive molecules, such as stem cells, to target treatments for wound-healing indicators such as angiogenesis, collagen deposition, and inhibition of inflammatory responses to treat chronic wounds.

The review has highlighted how hydrogel delivery of stem cells to the injury site is an effective strategy for improving chronic wound healing (Sivaraj et al., 2021). Stem cells, including MSCs, have the potential to undergo multidirectional differentiation, induce cell proliferation and migration to the wound surface, and promote skin repair (Nuschke, 2014). ECM and the wound-healing environment can also be regulated by synthesizing collagen and fibronectin or secreting growth factors (Badiavas and Falanga, 2003). Moreover, MSCs decrease the secretion of pro-inflammatory cytokines (tumor necrosis factor and interferon) and increase the expression of anti-inflammatory cytokines such as interleukin-10 (IL-10) and interleukin-4 (IL-4) (Na et al., 2017). They also act on fibroblasts and macrophages through GDF11 and TGF-β, mediating the immune response, cell proliferation, and angiogenesis (Sharma et al., 2021). VEGF and FGF are also important beneficial MSC mediators of angiogenesis (Werner, Grose). In addition, MSCs preferentially target injured or damaged tissue sites and are easily cultured *in vitro*, and many studies have used them in clinical applications (Shi et al., 2010). Hydrogels have good biocompatibility, degradability, adhesion, moisture resistance, and antimicrobial properties and can be used as a non-biological scaffold for delivering and continuously releasing stem cells. Bioactive hydrogel loaded with stem cells or stem cell-derived substances can significantly shorten wound healing time by inhibiting infection (Chen et al., 2021), reducing the inflammatory reaction (Garcia et al., 2019), stimulating cell migration and proliferation (Yu et al., 2020), and promoting angiogenesis in the wound healing process (Cerqueira et al., 2014).

Hydrogel-loaded stem cells for wound healing are expected to offer good clinical treatment (Supplementary Figure S1). However, there are few studies on the application of hydrogel stem cells in clinical practice, and the use of stem cells in human body is limited. In view of the characteristics of stem cells, the application of hydrogels is developing towards promoting the activity of stem cells, promoting the secretion of cytokines and the sustained release of stem cells. Moreover, hydrogels can be modified with macromolecules to obtain special properties suitable for chronic wounds, such as self-healing (Zhang et al., 2020), antioxidant (Xu et al., 2020), injectable (Gao et al., 2020), immunomodulatory (Kharaziha et al., 2021), and antibacterial hydrogels (Zhong et al., 2020). Furthermore, more distinctive hydrogel designs are emerging with the development of new synthetic and processing technologies. Currently, there exist many polymeric dynamic hydrogels that respond to changes in the cellular environment, enhancing the potential of stem cell-based wound therapy (Burdick et al., 2016).In addition, stem cell-derived exosomes are also an emerging therapeutic method that avoids the risk of directly introducing cells into the wound and has broad application prospects (Safari et al., 2022). Because the etiology of diabetic ulcer wounds is complex, future directions may include developing new hydrogel materials with multiple effects by combining multiple substances released at different wound healing stages (Bai Q et al., 2020). Conversely, burn research may instead focus on how to prevent scar formation, reduce patient pain (Wang et al., 2018), and develop skin graft substitutes.

However, pressure ulcers in DM patients are still a difficult problem to solve clinically. It has been shown that methylacrylic gelatin frozen hydrogel coated EPCs and acid fibroblast growth factor (aFGF) can treat pressure ulcers in DM rats (Zhu et al., 2022). Besides, there have been studies to promote the treatment of venous leg ulcers by wrapping antisense oligodeoxynucleotides targeting the mRNA of gap junction protein Cx43 in the thermoreversible hydrogel Pluronic F-127 (Gilmartin et al., 2016). In addition to applying hydrogel dressings, wound management strategies include several other common approaches. Compression therapy is the mainstay of venous ulcers, arterial ulcers may require revascularization surgery, and diabetic foot ulcers require ICC pressure relief (Alam et al., 2021). Additionally, hyperbaric oxygen therapy (HBOT) has physiological effects that can promote tissue repair and has multiple approved indications, but data on its treatment remain controversial. Studies suggest that HBOT is best used in diabetic foot ulcers, but its long-term efficacy remains to be determined (Kranke et al., 2015). Therefore, hydrogel combined with stem cells is a new and promising treatment method for chronic wounds.

This review helps to focus more precisely on the research progress of hydrogel combined with stem cells for wound healing (Supplementary Figure S2). Hydrogel dressings based on stem cell therapy for promoting wound healing are an important area for future research, including optimizing the hydrogel delivery system, maintaining loading material activity, and developing new active molecules to promote chronic wound healing. Moreover, the in-depth study of chronic wound mechanisms, including diabetic and deep burn wound healing, will enable us to expand the use of stem cell hydrogel therapy in treating chronic wounds.
