# Supplementary material for: Advances of hydrogel combined with stem cells in promoting chronic wound healing
Source: Front Chem. 2022 Nov 28;10:1038839. doi: 10.3389/fchem.2022.1038839 (PMC9742286; doi:10.3389/fchem.2022.1038839)
Supplement: Supplementary file 1 [file DataSheet1.pdf]

Supplementary Material

Supplementary Figures

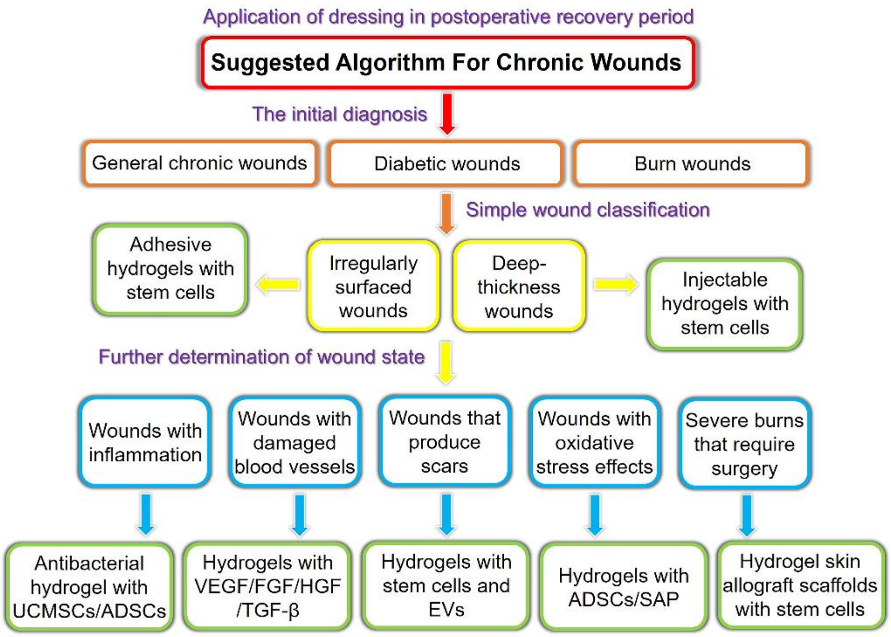

Supplementary Figure 1. Suggested algorithm for hydrogel-stem cells treatment to promote chronic wound healing.

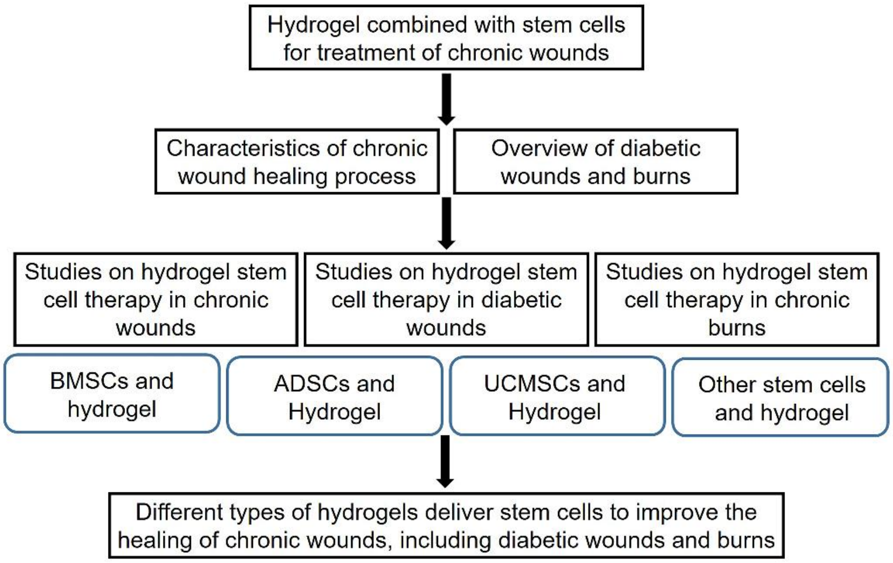

Supplementary Figure 2. Flow chart of the structure of the review.
